# Capital Punishment

**Published:** 1879-07

**Authors:** 


### CAPITAL PUNISHMENT.

It has always seemed to us, and always will, that the legal taking of a life is the poorest possible use that a criminal can be put to.

Perhaps it is necessary that life should be destroyed in order to intimidate would-be murderers. Likely, many murders are prevented by a wholesome fear of the gallows. But could not a punishment be devised that would be quite as much feared and yet put the murderer to some use?

Suppose the penalty for murder should be solitary confinement at hard labor during life, the criminal the while given the plainest food, but enough to sustain his strength. Then suppose the pardoning power should be withheld from those in authority; that a murderer once within the prison walls, remained there until death, and that his earnings should be used first to pay the expenses of his keeping, and the remainder paid to his family, if any, or if without family or relatives that he should naturally support, then have it go to the family of the murdered man.

It would seem as though that would be placing him to some good use. But when you hang him, he is utterly incapable of contributing to the aid of the family, either of the murdered person or his own, neither can he do the State any service.

Doubtless, there exist many criminals of whom it may be said "hanging is too good for them." We should like to see what effect hard labor, plain food and rigorous discipline would have upon them. Certainly they could do some good and no harm alive, while their death seems a much too easy way of settling the State's claim against them.

